# Assessment of Knowledge and Practices Toward COVID-19 Prevention Among Healthcare Workers in Tigray, North Ethiopia

**DOI:** 10.3389/fpubh.2021.614321

**Published:** 2021-06-23

**Authors:** Teferi G. Gebremeskel, Kalayu Kiros, Hailay A. Gesesew, Paul R. Ward

**Affiliations:** ^1^Department of Reproductive Health, College of Health Sciences, Aksum University, Aksum, Ethiopia; ^2^School of Medicine, College of Medicine and Health Science, Aksum University, Aksum, Ethiopia; ^3^Discipline of Public Health, Flinders University, Adelaide, SA, Australia; ^4^Department of Epidemiology, College of Health Sciences, Mekele University, Mekele, Ethiopia

**Keywords:** knowledge, perception, practice, COVID-19, healthcare workers, Tigray, Ethiopia

## Abstract

**Background:** The incidence rate of coronavirus disease 2019 (COVID-19) is increasing in several countries despite that public health measures are put in place. Given that COVID-19 is a newly emerging disease, there is little knowledge about the disease. The present study aims to assess knowledge, perception, and preventive practices toward COVID-19 among health workers in Tigray, North Ethiopia.

**Materials and Methods:** A health facility-based cross-sectional study was conducted among health professionals working in public hospitals. Data were collected between April and May 2020. The researchers included 403 participants and recruited them via a simple random sampling technique. To collect data, the researchers prepared a structured questionnaire guided by the WHO survey questions. Data were entered into Epi-info 7 and exported to SPSS version 20.00 for analysis. The researchers applied descriptive and inferential statistical analyses. Tables and graphs were used to describe data, and multivariate binary logistic regression was used to determine factors affecting knowledge, perception, and practices toward COVID-19 prevention.

**Results:** Among the participants, 79, 88, and 64.3% of them had adequate knowledge, positive perception, and good practice toward preventing COVID-19, respectively. Besides, 92% of the study participants knew that the COVID-19 virus does not have curative treatment and vaccine. The findings revealed that 55% of the respondents did not use the necessary personal protective equipment (PPE) at all times. The result showed that being female [AOR: 2.43, 95% CI (1.50–3.94)] and having a work experience of 2–5 years [AOR: 2.44, 95% CI (1.10–5.39)], news media as a source information [AOR: 7.11, 95% CI (3.07–16.49)], social media as a source information [AOR: 4.59, 95% CI (2.15–9.84)], and governmental website as a source information [AOR: 4.21, 95% CI (2.15–8.27)] were reported as protective factors; and being single [AOR: 0.15, 95% CI (0.03–0.75)] was reported as risk factor toward the prevention of COVID-19.

**Conclusion:** Most health workers had adequate knowledge and positive attitude toward COVID-19; nevertheless, a significant proportion of health workers had poor practice toward the prevention of COVID-19, including the use of PPE. Additionally, some groups of health professional showed poor practices of implementing the public health measures, hence the call for them to improve in the prevention and control of COVID-19.

## Introduction

Coronavirus disease 2019 (COVID-19) is currently a global health and public health emergency (1). The first outbreak of severe respiratory syndrome associated with coronavirus was first reported in 2003 (2). In December 2019, Wuhan, Hubei Province, China, became the center of an outbreak of pneumonia of unknown cause, which was later known as a novel COVID-19 (3, 4). The COVID-19 burden has increased around the world in disease, death, and economic crises (5). Globally, there are 31,867,173 infections and 967,258 deaths on September 24, 2020 (6). Africa has recorded 1,420,629 cases and 34,327 deaths as of September 24, 2020 (6). The first reported coronavirus patient in Ethiopia, a Japanese citizen, was observed on March 13, 2020 (7). There are more than 71,083 cases and 1,141 deaths in Ethiopia on September 23, 2020 (8). Tigray reported 5,316 cases and 27 deaths as of September 23, 2020 (9).

Several countries put public health measures in place to control the transmission of COVID-19. Nevertheless, the compliance with these measures is not at the desired level. Some of the reasons include long-term vulnerability, lack of personal protective equipment (PPE), overcrowding, lack of isolation facilities, contaminated environment, and possibly a lack of knowledge and understanding among healthcare workers (HCWs) (10–12).

HCWs are at high risk for COVID-19 because of the nature of their work, which exposes them to infectious diseases on a daily basis. Worldwide, many HCWs are infected with the COVID-19 and have lost their lives (5, 13). Unless special attention is paid to the safety of HCWs and their workplaces, the system will lose many HCWs and severely undermine the capacity of anti-COVID-19 and other infectious diseases worldwide. Unlike other people, HCWs have a double source of COVID-19 in their community and workplaces. The main cause include long-term vulnerability, lack of PPE, overcrowding, lack of isolation facilities, contaminated environment, and possibly a lack of knowledge and understanding among HCWs (11, 14). HCWs are a common source of family, patient, and community infections (12, 14).

The World Health Organization (WHO) recommends the prevention of COVID-19 transmission by maintain social distancing (at least 1 m) from any person by avoiding close contacts, hand hygiene (wash with soap or using alcohol-based hand sanitizers), and wearing PPE (15, 16). The WHO also launched a number of online training courses and materials on COVID-19 in different languages to facilitate the preventive mechanisms, including increasing awareness and capacity building HCWs in preparation activities (17). Often, misunderstandings among HCWs have slowed down efforts to provide the necessary treatment, which has led to the rapid spread of infection in hospitals and has endangered patients' lives (18, 19).

It is also important to improve the knowledge and prevention practice of HCWs and the community through regular updates on COVID-19 (15, 19). If HCWs have access to information, they will improve their knowledge, implement preventive devices to COVID-19, and provide better care for patients, families, and the community (11, 20).

Studies showed that HCWs have 93.2% good knowledge, 95% positive attitude, and 88.7% good practice regarding COVID-19 (21). Reports from the healthcare professionals in Greece toward severe acute respiratory syndrome coronavirus 2 (SARS-CoV-2) showed that 88.3% of the subjects had good knowledge and 71% of the participants agreed to temporary travel restrictions (22). Cross-sectional studies in Egypt showed that the average correct answer for COVID-19 prevention-related questions was 80.4% with a mean knowledge score of 18.5 ± 2.7 out of 24. About 83.1% of the participants feared COVID-19, and 89.2% said they had a higher risk of COVID-19 than others (23). Additionally, there are different works on knowledge and practices on COVID-19 in Ethiopia and Africa (24–27).

There is an inadequate study of COVID-19 prevention practice of HCWs in Ethiopia in general and in Tigray in particular. Early prevention of the disease before its entry has paramount importance. The level of knowledge, perception, and preventive practice of health workers is indispensable to successful early prevention of the disease. Thus, this research paper is supposed to fill the gap by identifying the status of knowledge, perception, and preventive practice of HCWs toward COVID-19. Besides, the study will be helpful by shedding light on intervention areas that need to be pursued by policymakers. This study aims to assess the knowledge, perception, and practice of HCWs about COVID-19 in Aksum University Comprehensive and Specialized Hospital (AKUCSH) and Saint Mary's General Hospital in Tigray Regional State of Northern Ethiopia.

Based on these considerations, therefore, the following hypotheses were formulated:

1. Age, gender, work experience, news media, social media, governmental website, family, and friends as source of information would significantly predict knowledge of COVID-19 among health workers.
2. Age, ethnicity, news media, social media, governmental website, family, and friends as source of information would significantly predict perception of COVID-19 among health workers.
3. Age, gender, marital status, news media, family, and friends as source of information would significantly predict practices of COVID-19 prevention among health workers.

## Materials and Methods

### Study Design, Setting, and Population

A health facility-based cross-sectional study design was employed among health workers. The study was conducted in AKUCSH and Saint Mary's General Hospital in Tigray Regional State of Northern Ethiopia. Axum city is located 1,045 km away from Addis Ababa, the capital city of Ethiopia, and 262 km from Mekele capital city of Tigray regional state. Axum city has five kebeles (a small administrative unit consisting of 20,000 population), one referral and teaching hospital, one general hospital, two health centers, four health posts, and 10 different level private clinics. AKUCSH provides curative and preventive services and has 330 HCWs including 22 specialists, 84 general practitioners (GPs), 218 nurses, and six health officers. AKUCSH provides health services for 3.6 million people on average. Saint Mary's General Hospital, the other study setting, was established in 1961 and has 258 HCWs including six specialists, 17 GPs, 212 nurses, eight health officers, seven pharmacists, and eight laboratory technicians.

HCWs, including physicians, pharmacists, nurses, and laboratory technicians, who have a work experience of 6 months, were eligible to be included in the study. HCWs who were on annual leave and were not willing to participate were excluded.

### Sample Size and Procedure

The sample size was determined using the formula of the single population proportion. The following parameters were used to calculate the sample size: p = proportion HCWs who are knowledgeable about COVID-19 [50%, no previous study found in Ethiopia; 95% CI (Z_1−α/2_) = 1.96], and 5% degree of marginal error (d). Assuming a 5% non-response rate, the minimum required sample size was 403. A simple random sampling technique was employed to recruit study participants.

### Variables and Measurements

The knowledge, perception, and practice toward COVID-19 prevention were measured based on the WHO (2020) Survey Tool and guidelines for National comprehensive COVID 19 management Federal ministry of Health (FMOH), Ethiopia (28). The questions about the knowledge of COVID 19 prevention had 15 items, the questions about the respondents' perception toward COVID 19 prevention had 11 items, and the questions about the respondents' practice toward COVID 19 prevention had 10 items. The rest of the questions were about the respondents' sociodemographic information. All the questions contained the categories “yes,” “no,” and “don't know.” The respondent's knowledge toward COVID 19 prevention was indicated by two categories: “Inadequate knowledge” for <9 of 15 items (<60%) and “adequate knowledge” for ≥9 of 15 items (≥60%) (27, 29, 30). The respondent's perception toward COVID-19 was indicated by two categories: “negative perception” for <7 of 11 items (≤60%) and “positive perception” for ≥7 of 11 items (>60%) (27, 29, 30); a reliability coefficient (Cronbach's alpha) of 0.60 was obtained in a pilot testing of the scale, while the current data set yielded 0.65. Especially regarding the practice toward COVID-19, the respondents were asked about going to crowded places, wearing masks in public, maintaining social distance, hand washing, avoiding handshaking, and obeying government restrictions. The respondents' practices toward COVID-19 prevention were indicated by two categories: “poor practice” for <5 of 10 items (<50) and “good practice” for ≥5 of 10 items (≥50%) (27, 29, 30).

### Data Collection Process and Quality Assurance

Data were collected from April to May 2020, via an interview with a pretested and structured questionnaire used from the WHO survey questions. The questionnaire includes sociodemographics, disease knowledge, disease perception, and preventive practices. The data extraction sheet was prepared in English, then translated into the local language (Tigrigna), and translated back into English by a professional. To establish face validity and translation quality, the questionnaire was tested on 5% of the total sample size outside of the study site by data collectors and supervisors. A few questions, language clarity, and information were revised; and the questionnaire was finalized for the study by the principal investigators. Three data collectors and supervisors were recruited outside of the study site, and they were given training for 2 days. The supervisors supervised the process of data collection, checked the data completeness consistency, and communicated with principal investigators daily.

### Data Analysis

After being coded, data were entered into Epi-info 7 and exported to SPSS version 20.00 for analysis. Simple descriptive statistics such as frequency, percentage, and mean were employed. Tables, charts, and graphs were used to present the result of the analyzed data. A binary logistic regression model was used to determine sociodemographic factors predicting knowledge, perception, and practice toward prevention and control of COVID-19. Variables with *p* < 0.2 were recruited for multivariate logistic regression analysis. Adjusted odds ratio with 95% of CI was calculated, and *p* < 0.05 were considered as a cutoff for the statistically significant association.

### Ethics Statement

Ethical clearance was obtained on June 19, 2020, from the Institutional Review Committee (IRC) (IRB Number: IRB I79/2020) of the College of Medicine and Health Sciences, University of Aksum. A permission letter was received from those administrative bodies of the health facilities. Written consent was obtained from every study participant included in the study during data collection time after the objectives of the study and the right to withdraw from the study at any time were explained. The data were kept confidential, and the results did not identify the respondents personally.

## Results

A total of 403 health workers were included in the study, with a response rate of 96%. The mean age of the study participants was 28.2 + 5.1 years, with a minimum and maximum age of 19 and 46 years, respectively. More than half (51.7%) of the participants were male, and the majority of the participants (52.7%) were single. In terms of profession, 54% of participants were nurses followed by 20.7% physicians, 10.6% midwives, 9% pharmacists, and 5.7% laboratory technicians. The majority of the participants (83.2%) were ethnic Tigray. Table 1 describes the demographic characteristics of the study participants.

**Table 1:** Sociodemographic characteristic of health workers at Aksum University Comprehensive and Specialized Hospital (AKUCSH) and Saint Mary's General Hospital in Tigray Regional State of Northern Ethiopia (*n* = 403), April to May 2020.

**Characteristics**		**Frequency**	**Percent**
Age	≤25	104	26.9
	26–29	143	37.0
	>30	140	36.2
Sex	Male	200	51.7
	Female	187	48.3
Marital status	Single	204	52.7
	Married	176	45.5
	Divorced	7	1.8
Religion	Orthodox	372	96.1
	Muslim	13	3.4
	Protestant	2	0.5
Ethnicity	Tigray	322	83.2
	Amara	57	14.7
	Oromo	8	2.1
Profession	Physician	80	20.7
	Nurse	209	54.0
	Midwifery	41	10.6
	Pharmacy	35	9.0
	Laboratory technician	22	5.7
Work experience in the year	<2 years	157	40.6
	2–5 years	143	37.0
	≥5 years	87	22.5
Have heard about COVID-19	Yes	386	99.7
	No	1	0.3
Training on COVID-19	Yes	273	70.5
	No	114	29.5

### Knowledge, Perception, and Practice of Healthcare Workers Toward COVID-19

**Figure 1:**
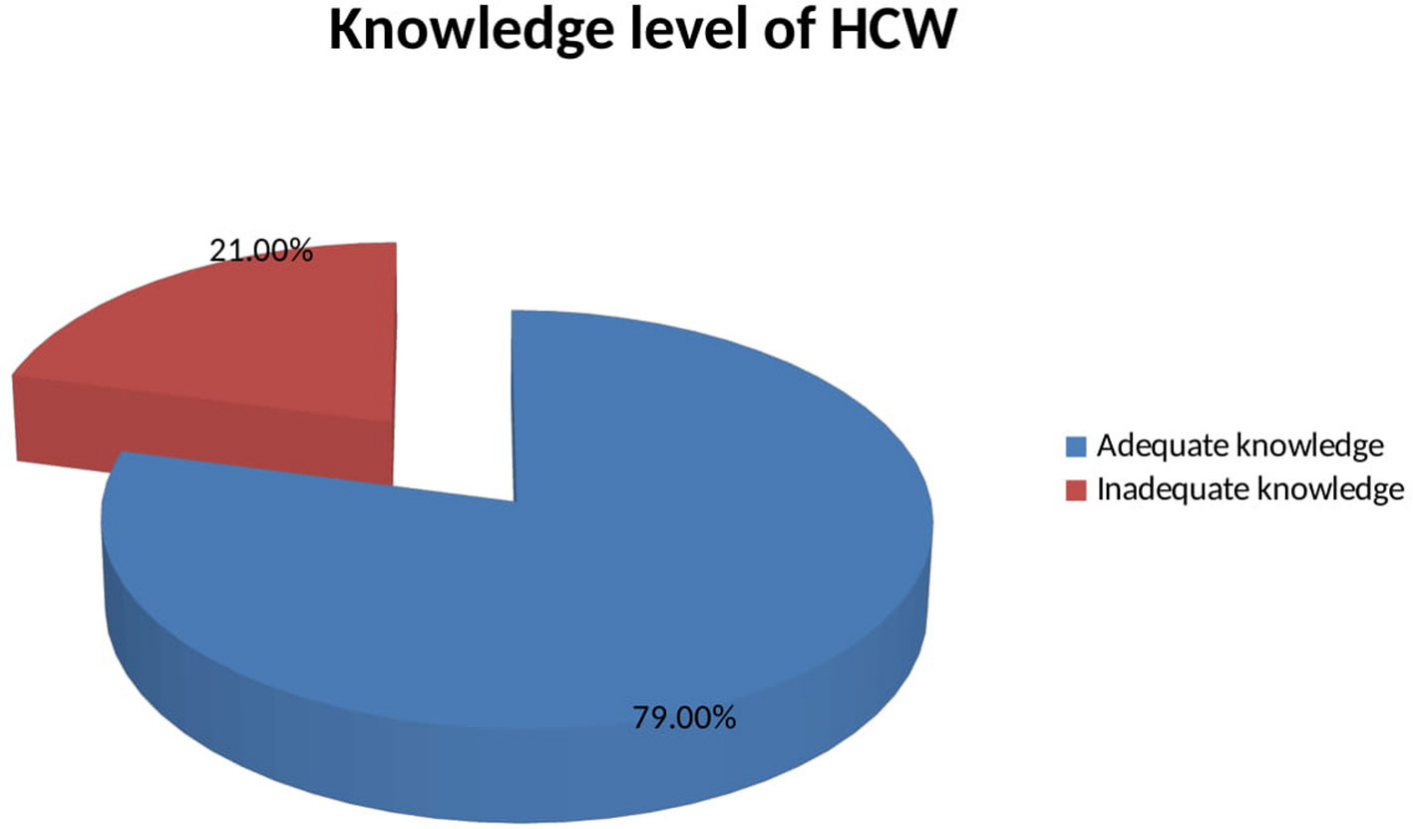
Knowledge of COVID-19 among health workers at Aksum University Comprehensive and Specialized Hospital (AKUCSH) and Sain't Marry's General Hospital in Tigray Regional State of Northern Ethiopia.

**Figure 2:**
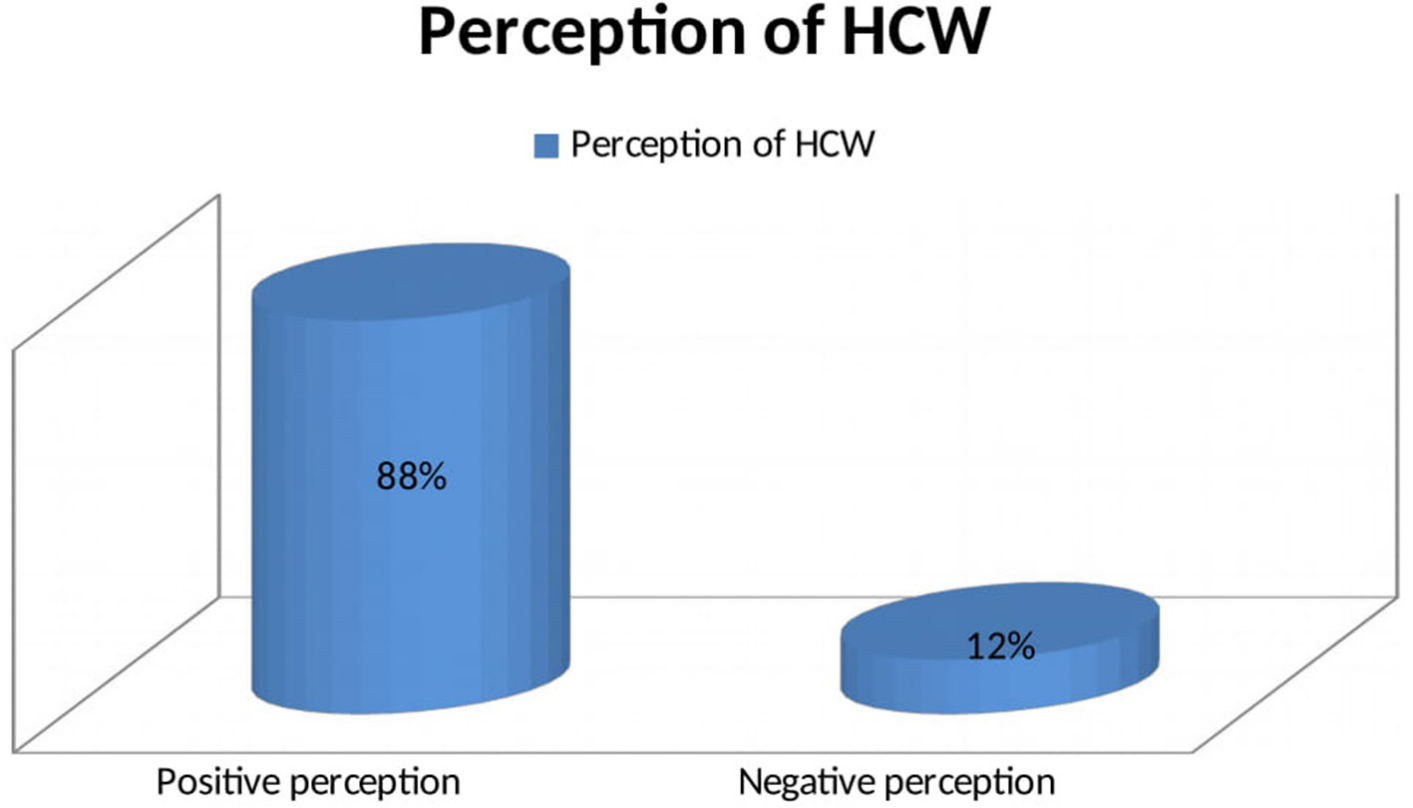
Perception of COVID-19 among health workers at Aksum University Comprehensive and Specialized Hospital (AKUCSH) and Sain't Marry's General Hospital in Tigray Regional State of Northern Ethiopia.

**Figure 3:**
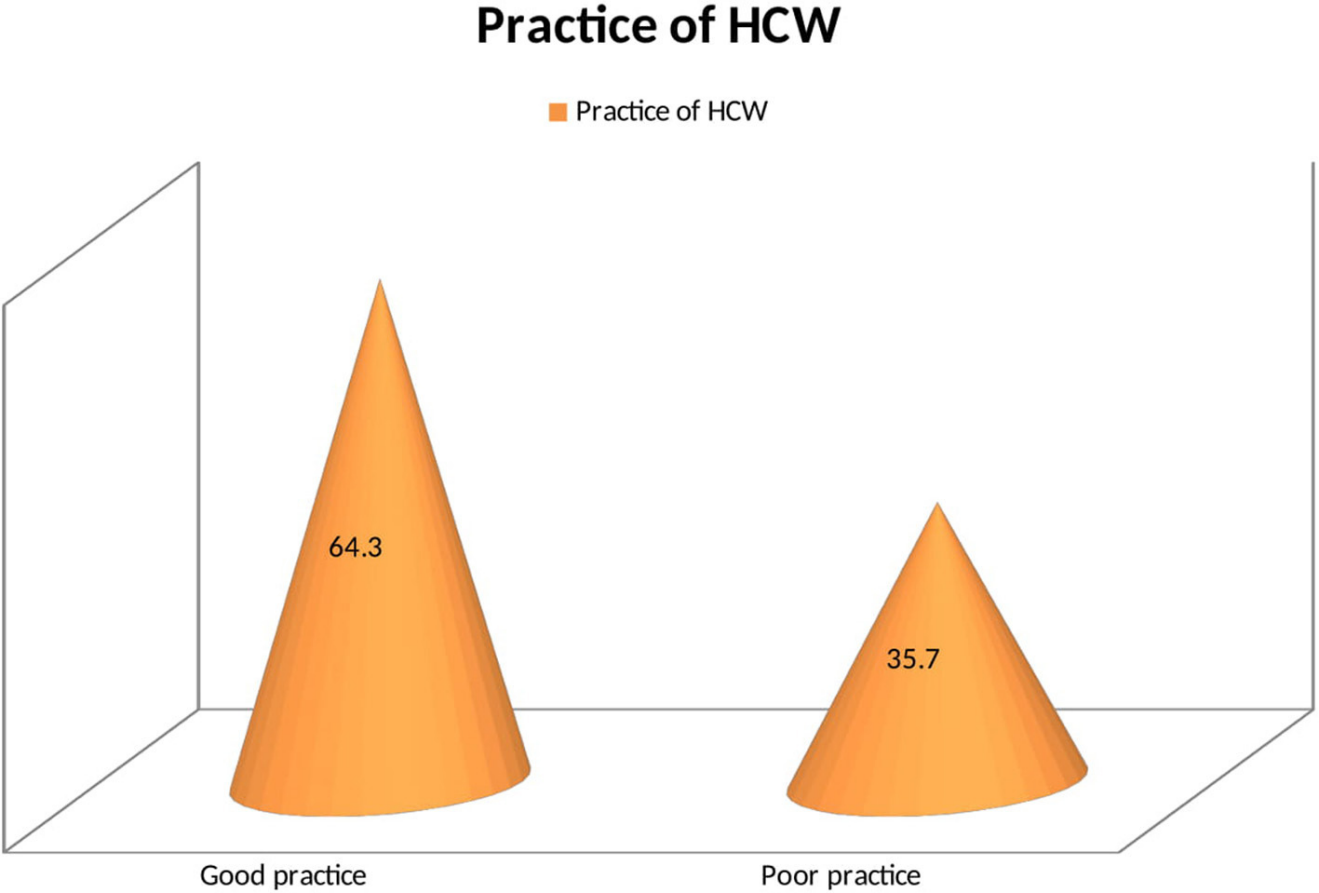
Practice of COVID-19 prevention among health workers at Aksum University Comprehensive and Specialized Hospital (AKUCSH) and Sain't Marry's General Hospital in Tigray Regional State of Northern Ethiopia.

More than three fourths (79%) of health workers were knowledgeable about COVID-19 (Figure 1). The majority of participants (92%) knew that the COVID-19 has no special treatment and vaccine (Figure 1). Likewise, 87.9% of HCWs had a positive perception of COVID-19. Almost all participants (97.4%) perceived that washing hands with soap and water was the best prevention of COVID-19 (Figure 2). More than half (64.3%) of health workers had good practice toward COVID-19 prevention (Figure 3). Almost all of the health workers (96.1%) kept their hand hygiene (wash with soap or using alcohol-based hand sanitizers). More than half of health workers (54.8%) did not use the necessary PPE at all times (Figure 3).

### Factors Associated With Knowledge

Age, gender, work experience in years, news media as source of information, social media, governmental website, family, and friends were included in the multivariable analysis. In the multivariable logistic regression analysis, work experience and governmental website as source of information were significantly statistically associated with adequate knowledge of COVID-19. Participants with work experience between 2 and 5 years were two times [AOR: 2.44, 95% CI (1.10–5.39)] more likely to be knowledgeable than participants with ≥5 years of work experience. Participants having a governmental website as a source information were four times [AOR: 4.21, 95% CI (2.15–8.27)] more likely to be knowledgeable about COVID-19 prevention than those who were not (Table 2).

**Table 2:** Univariate and multivariate logistic regression analyses of knowledge about COVID-19 among health workers at Aksum University Comprehensive and Specialized Hospital (AKUCSH) and Saint Mary's General Hospital in Tigray Regional State of Northern Ethiopia (*n* = 403), April to May 2020.

**Variable**		**COVID-19 knowledge**		**COR**	**AOR**
		**Adequate**	**Inadequate**		
Age	≤25	34 (32.7)	70 (67.3%)	2.13 (1.18–3.85)^*^	1.79 (0.83–3.89)^*^
	26–29	20 (14%)	123 (86%)	0.71 (0.38–1.35)^*^	0.61 (0.30–1.23)^*^
	>30	26 (18.6%)	114 (81.4%)	1	1
Sex	Male	167 (83.5)	33 (16.5)	1	1
	Female	140 (74.9)	47 (25.1)	0.59 (0.39–0.97)^*^	0.68 (0.39–1.19)^*^
Work experience in the year	<2 years	32 (20.4%)	125 (79.6%)	1.46 (0.72–2.95)^*^	1.66 (0.67–4.13)^*^
	2–5 years	35 (24.5%)	108 (75.5%)	1.86 (1.09–3.72)^*^	2.44 (1.10–5.39)^*^
	≥5 years	13 (14.9%)	74 (85.1%)	1	1
News media as a source information	Yes	278 (80.8%)	66 (19.2%)	2.03 (1.01–4.06)^*^	1.60 (0.74–3.48)^*^
	No	29 (67.4%)	14 (32.6%)	1	1
Social media as a source information	Yes	245 (82.2%)	53 (17.8%)	2.01 (1.17–3.46)^*^	1.73 (0.91–3.27)^*^
	No	62 (69.7%)	27 (30.3%)	1	1
Governmental website as a source information	Yes	182 (89.2%)	22 (10.8%)	3.84 (2.24–6.59)^*^	4.21 (2.15–8.27)^**^
	No	125 (68.3%)	58 (31.7%)	1	1
Family and friends as a source information	Yes	146 (82.5%)	31 (17.5%)	1.43 (0.87–2.37)^*^	0.58– (0.29–1.14)^*^
	No	161 (76.7%)	49 (23.3%)	1	1

*COR, crude odds ratio; AOR, adjusted odds ratio*.

* *p < 0.05*;

** *p < 0.001*.

### Factors Associated With Perception

Age, news media, social media, governmental website, family, and friends as source of information were included in the multivariable analysis. In the multivariable logistic regression analysis, news media and social media as source of information were significantly associated with a positive perception of COVID-19. Participants having news media as source of information were seven times [AOR: 7.11, 95% CI (3.07–16.49)] more likely to have a positive perception toward COVID-19 than participants who did not attend news media. Those with exposure to social media [AOR: 4.59, 95% CI (2.15–9.84)] was found have a positive perception than those with non-exposure to social media (Table 3).

**Table 3:** Univariate and multivariate logistic regression analyses of perception about COVID-19 among health workers at Aksum University Comprehensive and Specialized Hospital (AKUCSH) and Saint Mary's General Hospital in Tigray Regional State of Northern Ethiopia, (*n* = 403), April to May 2020.

**Variable**		**COVID-19 perception**		**COR**	**AOR**
		**Positive**	**Negative**		
Age	≤25	92 (88.5%)	12 (11.5%)	0.70 (0.33–1.49)^*^	0.74 (0.31–1.76)^*^
	26–29	130 (90.9%)	13 (9.1%)	0.53 (0.26–1.11)^*^	0.59 (0.26–1.37)^*^
	>30	188 (84.3%)	22 (15.7%)	1	1
News media as a source information	Yes	316 (91.9%)	28 (8.1%)	8.94 (4.37–18.27)^**^	7.11 (3.07–16.49)^**^
	No	24 (55.8%)	19 (44.2)	1	1
Social media as a source information	Yes	278 (93.3)	20 (6.7%)	6.05 (3.19–11.49)^**^	4.59 (2.15–9.84)^**^
	No	62 (69.7%)	27 (30.3%)	1	1
Governmental website as a source information	Yes	191 (93.6%)	13 (6.4%)	3.35 (1.71–6.58)^**^	1.53 (0.66–3.54)^*^
	No	149 (81.4%)	34 (18.6%)	1	1
Family and friends as a source information	Yes	170 (96%)	7 (4%)	5.71 (2.49–13.11)^**^	2.25 (0.81–6.29)^*^
	No	170 (81%)	40 (19%)	1	1

*COR, crude odds ratio; AOR, adjusted odds ratio*.

* *p < 0.05*;

** *p < 0.001*.

### Factors Associated With the Practice

Age, gender, marital status, news media as source of information, and family and friends as source of information were included in the multivariable analysis. In the multivariable logistic regression analysis, gender and marital status were significantly statistically associated with good knowledge of COVID-19. Males were two times [AOR: 2.43, 95% CI (1.50–3.94)] more likely to have good practice to prevent COVID-19 than females. The odds of reporting good practice to prevent COVID-19 were lower among single participants compared with divorced participants [AOR: 0.15, 95% CI (0.03–0.75)] (Table 4).

**Table 4:** Univariate and multivariate logistic regression analyses showing predictors of practice to prevent COVID-19 among health workers at Aksum University Comprehensive and Specialized Hospital (AKUCSH) and Saint Mary's General Hospital in Tigray Regional State of Northern Ethiopia (*n* = 403), April to May 2020.

**Variable**		**Practice toward COVID-19**		**COR**	**AOR**
		**Good**	**Poor**		
Age	≤25	70 (67.3%)	34 (32.7%)	0.71 (0.42–1.20)^*^	1.34 (0.71–2.53)^*^
	26–29	96 (67.1%)	47 (32.9%)	0.71 (0.44–1.16)^*^	0.86 (0.51–1.45)^*^
	>30	83 (59.3%)	57 (40.7%)	1	1
Sex	Male	111 (55.5%)	89 (45.5%)	2.26 (1.47–3.47)^**^	2.43 (1.50–3.94)^**^
	Female	138 (73.8%)	49 (26.2%)	1	1
Marital status	Single	138 (67.6%)	66 (32.4%)	0.36 (0.08–1.65)^*^	0.15 (0.03–0.75)^*^
	Married	108 (61.4%)	68 (38.6%)	0.47 (0.10–2.18)^*^	0.26 (0.05–1.28)^*^
	Divorced	3 (42.9%)	4 (57.1%)	1	1
News media as a source information	Yes	227 (66.0%)	117 (44.0%)	1.85 (0.98–3.51)^*^	1.65 (0.81–3.36)^*^
	No	22 (51.2%)	21 (48.8%)	1	1
Family and friends as a source information	Yes	119 (67.2%)	58 (32.8%)	1.26 (0.83–1.92)^*^	1.13 (0.71–1.80)^*^
	No	130 (61.9%)	80 (38.1%)	1	1

*COR, crude odds ratio; AOR, adjusted odds ratio*.

* *p < 0.05*;

** *p < 0.001*.

## Discussion

This study aimed to assess knowledge, perception, and practice toward the prevention and control of the COVID-19 outbreak among HCWs in Northern Ethiopia. The finding showed that the majority of health workers had adequate knowledge (79%) of COVID-19. This finding is consistent with findings from other studies in North Ethiopia, 74% (27). The knowledge level of prevention and control of the COVID-19 outbreak in our study were lower than those of the cross-sectional study conducted in Iran, 85%; Henan China, 89%; and Pakistan, 93.2% (21, 31, 32). But these findings are higher than the knowledge level of prevention and control of the COVID-19 outbreak seen in studies conducted in Saudi Arabia, 51% (33); and Iran, 61% (34). The difference could be due to the frequency and focus of presentation of COVID-19 in media and the public in these countries. The commitment and leadership of the government to give focus on informing the public about the pandemic may also be another reason for the difference. This builds on the finding on the source of knowledge about COVID-19, which is similar to the study conducted on China residents (29). Most HCWs get information from the news media (89%) and social media (77%) about the COVID-19. Interestingly, this finding differs from that in the study in Saudi Arabia, which indicates that the ministry of health website is one of the main sources of information (33). These findings implied that the Ethiopian Government and the Ministry of Health need to plan health education programs about this COVID-19 outbreak.

Several variables predicted the level of knowledge regarding COVID-19 in our setting. Participants with work experience of between 2 and 5 years were two times more likely to be knowledgeable than participants with ≥5 years of work experience. This is because mobile internet and social media or technology (Facebook, YouTube, Telegram, and Twitter) are easily accessible by most health professionals at home and in the workplace. Participants having a governmental website as a source information was four times more likely to be knowledgeable those who were not, which is in agreement with a study conducted in Saudi Arabia where most health workers have access to information about COVID-19 and other infectious diseases through the Ministry of Health website (33, 35). This implied that the Ethiopian Government and the Ministry of Health need to use the governmental website to disseminate information to HCWs.

In this study, the majority (87.9%) of health workers had a positive perception of COVID-19. This finding is higher than the findings from other studies in North Ethiopia, 74% (27); Saudi Arabia, 51% (33); and Iran, 61% (34). The difference could be due to the frequency and focus of presentation of COVID-19 in media and the public in these countries. Almost all participants (97.4%) perceived that washing hands with soap and water were the best COVID-19 prevention. Majority of HCWs, 89.4%, recognized that COVID-19 is a fatal disease. This study also revealed that participants having news media as a source of information and exposure to social media were associated with positive perception of COVID-19. This is consistent with other studies where social media, if used wisely, can serve as a powerful tool to change people's behavior and improve the health of individuals and nations (36). This is because mobile internet and social media or technology (Facebook, YouTube, Telegram, and Twitter) are easily accessible by most health professionals at home and in the workplace. This implied that the Ethiopian Government and the Ministry of Health need to use news and social media to disseminate information to HCWs.

More than half (64.3%) of health workers had good practice toward COVID-19 prevention. Almost all of the health workers (96.1%) kept hand hygiene (wash with soap or using alcohol-based hand sanitizers) consistently. This is similar to the findings of studies conducted in China and the United Arab Emirates (29, 32, 37, 38). More than half of health workers (54.8%) did not use the necessary PPE at all times, maybe due to lack of PPE, not comfortable using the PPE, negligence, lack of safety and health education, and lack of knowledge and practice. Males were two times more likely to have good practice to prevent COVID-19 than females. Interestingly, this finding differs from those previous findings: a significant association between male gender and potentially dangerous practices toward COVID-19 was found in this study (29, 39–41). This is because stay-at-home orders also make it difficult for many women to procure food for cooking, one of their key responsibilities directly affected by COVID-19. Some women will need to decide to spend time outside the home to procure either safe water or food for their children and families. And food insecurity may affect women more than men, as seen in a study from Ethiopia (42).

The study has the following limitations. First, findings from a cross-sectional study design could not confirm the cause-and-effect relationship. Second, there may be an information bias given the collected data were self-reported. Third, health workers were the study participants; and the level of knowledge, perception, and practice may be different from that of the public.

## Conclusions

Most health workers have adequate knowledge; nevertheless, a significant proportion of health workers had poor practice toward the prevention of COVID-19, including the use of PPE. Additionally, some groups of health professional had poor practices of implementing the prevention and control of COVID-19, hence the call for them to improve in the prevention and control of COVID-19. The majority of health workers do not use the necessary PPE at all times. Work experience, governmental website as a source information, and sex were protective factors; and ethnicity and marital status were risk factors toward prevention and control of COVID-19. These imply target areas and groups of HCWs to focus on preventing the spread of the coronavirus. We recommend for researchers to conduct qualitative study and to include the variables that could not be addressed using a cross-sectional study design.

## Data Availability Statement

The data supporting the conclusions of this article are included in the article. The raw data supporting the conclusions of this article will be made available by the authors, without undue reservation, to any qualified researcher.

## Ethics Statement

The studies involving human participants were reviewed and approved by Ethical clearance was obtained from the Institutional Review Committee (IRC) of the College of Medicine and Health Sciences, University of Aksum. A permission letter was received from those administrative bodies of the health facility's verbal. Written consent was obtained from all participants after they were informed on the purpose of the study. The patients/participants provided their written informed consent to participate in this study.

## Author Contributions

TG and KK designed the study. TG performed statistical analyses and drafted the manuscript. All authors contributed to writing the manuscript, read, and approved the final manuscript.

## Conflict of Interest

The authors declare that the research was conducted in the absence of any commercial or financial relationships that could be construed as a potential conflict of interest.

## Abbreviations

CDC: Centers for Disease Control and Prevention
COVID-19: coronavirus disease 2019
MERS: Middle East respiratory syndrome
SARS: severe acute respiratory syndrome
SARS-CoV-2: severe acute respiratory syndrome coronavirus 2
WHO: World Health Organization
CI: confidence interval
KAPs: Knowledge, Attitude and Practices
OR: odds ratio
HCW: healthcare worker.
