# Supplementary material for: Assessment of Knowledge and Practices Toward COVID-19 Prevention Among Healthcare Workers in Tigray, North Ethiopia
Source: Front Public Health. 2021 Jun 23;9:614321. doi: 10.3389/fpubh.2021.614321 (PMC8260836; doi:10.3389/fpubh.2021.614321)
Supplement: Supplementary file 1 [file Data_Sheet_1.PDF]

Questionnaire format for Coronavirus 2019 (COVID-19)

More than one answer is possible for MCQ

I. Identification

1. Questionnaire I.D: _______________

II. Socio-demographic characteristics

1. Sex A. Male B. Female
2. Age in year_________
3. Religion
   A. Orthodox's B. Muslim C. Protestant D. Catholic E. Others specify---------
4. Marital status
   A. married B. Single C. Divorced D. widowed E. if other specify_________
5. Ethnicity
   A. Tigray B. Amharic C. Oromo D. SNNP E. other specific__________
6. Occupation
   A. Medical doctors B. Medical interns C. Nurse D. Midwife E. Pharmacy F. Laboratory G. Cleaner H. Porter F. other _______
7. Work experience in years _______
8. Work place (current) A. OPD B. Emergency C. Ward D. ICU E. laboratory F. pharmacy G. cleansing service

III. Awareness about COVID-19 (more than one answer is possible)

1. Have heard about novel corona virus (COVID-19) A. Yes B. NO
2. If your answer is yes for Question number 1 from where do get the reliable information
   A. news media (TV/video, magazines, newspapers, and radio)
   B. social media (Face book, Twitter, Whatsapp, YouTube, Instagram)
   C. official government websites
   D. family and friends
   E. Other _________
3. Have you attended lectures, meetings or discussions about Novel Corona virus
   A. Yes B. NO

I. Knowledge about COVID-19 (more than one answer is possible)

1. What is the causative agent of COVID-19
   A. Virus B. Insect bite C. GOD curse or Anger D. other ______
2. From where does COVID-19 is originated from
   A. Human B. Bats C. Camel D. other_______
3. What is (are) the transmission route (s) of COVID-19
   A. Direct contact
   B. Feco-oral
   C. Airborne
   D. using of equipments come from abroad
   E. insect bite
   F. Eating of undercooked meats
   G. Via droplets while coughing or sneezing
   H. other _______

4. What are the clinical manifestations of COVID-19
   A. fever B. cough C. sore throat D. shortness of breath E. headache F. myalgia G. Arthralgia H. fatigue I. chills and rigors J. others _______
5. What is the incubation period of COVID-19?
   A. < 2 days B. 2-14 days C. 14-30 days D. other _______
6. What are the complications of COVID-19?
   A. Severe pneumonia B. Acute Respiratory Disease Syndrome (ARDS) C. Sepsis and septic shock D. Multiorgan failure E. death F. other _______
7. What is the confirmatory diagnosis of COVID-19?
   A. Based on clinical sign and symptoms B. Chest X-ray C. CT scan D. laboratory E. other _____
8. What is the current treatment option of COVID-19?
   A. Supportive care B. Antimicrobials C. Antiviral D. Antifungals E. other _______ F. No drug treatment available
9. Does COVID-19 have a vaccination currently?
   A. Yes if yes specify _______ B. No
10. Which race does COVID-19 mostly affect?
    A. White B. Black C. No racial difference D. I don't know
11. Which age group does COVID-19 affect?
    A. Children B. Adolescents C. Adults D. Old ages E. affect all ages
12. What are the vulnerable groups (at risk of morbidity and mortality) of COVID-19?
    A. Children B. Old age C. Males D. DM patient E. Cardiac Patient F. those who have Cancer G. other _______
13. What are the prevention methods of COVID-19 transmission
    A. Hand hygiene (wash with soap or alcohol rub)
    B. covering nose and mouth while coughing or sneezing
    C. avoiding sick contacts
    D. Social distancing (at least 1 meter) from person who have COVID-19 sign and symptoms
    E. Social distancing (at least 1 meter) from any person
    F. Stay at home
    G. Vaccination
    H. Repeatedly drinking water
    I. Garlic and other traditional medicines
    J. Avoid use of equipments come from abroad
    K. Taking hot shower
    L. other _______
    M. no preventive measure

14. Who COVID-19 patients are candidate for hospital intervention (admissions)
    A. All patients
    B. mild illness
    C. Severe and complicated
    D. Pregnant
    E. other _______
15. Who should use face mask?
    A. Person who has fever, cough, shortness of breath or sneezing
    B. Person who had contact with patient who has COVID-19 symptoms
    C. Confirmed COVID-19 patient
    D. Person who work on health facility or get service from health facility
    E. Healthy person
    F. Other _______

II. Perception of Healthcare workers' towards COVID-19 (more than one answer is possible)

1. Do you think COVID-19 is fatal A. yes B. NO
2. Do you think flu vaccination is sufficient to prevent COVID-19 A. yes B. NO
3. Do you think sick patients should share their recent travel history A. yes B. NO
4. Do you think COVID-19 symptoms appear in 2-14 days A. yes B. NO
5. Do you think washing hands with soap and water could help in prevention of COVID-19 transmission A. yes B. NO
6. Do you think disinfect equipment's and working area in wet markets at least once a day is necessary A. yes B. NO
7. During the outbreak, eating well-cooked and safely handled meat is safe A. yes B. NO
8. What concerns you most about coronavirus?
    A. Global infection B. Local infection C. Economic impact D. Travel restriction E. Contacting the disease myself F. other ___
9. What do you do if you get a person having sign and symptoms of COVID-19?
    A. Advice to seek medical treatment from health institution
    B. Advice on home treatment
    C. Advice to wear mask
    D. Far from him/her to prevent myself
    E. Advice to go for traditional medicine
    F. Advice to use garlic and other private medicines
    G. Other ___
10. What do you think you should do yourself to prevent from COVID-19?
    A. Avoid crowded places (like sport center, Night clubs, hotels)
    B. Social distancing from any person at least 1 meter
    C. Opening windows of public transportation cars
    D. Limit number of person while public transportation
    E. Other ___

11. Where do you think you are at the greatest risk of exposure to coronavirus?
    A. Public places B. public transportation
    C. Hospital D. Home E. other ___

III. Practice of Healthcare workers' towards COVID-19 (more than one answer is possible)

1. Do you keep your hand hygiene (wash with soap or alcohol rub) A. yes B. NO
2. If Q1 Yes when
    a. Before using glove
    b. After using glove
    c. After touching any equipment
    d. before and/or after meal
    e. before and/or after touch mouth, nose and eye
    f. before entering to work place
    g. After leaving/exit to work place
3. Do you covering your nose and mouth while coughing or sneezing A. yes B. NO
4. Do you promote the adoption of safe working practices A. yes B. NO
5. Do you promote safe distance in triaging and management of cases A. yes B. NO
6. Do you apply safe transport of suspects to treatment and quarantine centers
   A. Yes B. No
7. Do you use all the necessary PPEs at all times? (observe) A. yes B. NO
8. If yes Q7 which do you use/apply
    a. Hand glove
    b. mouth masks
    c. Eye goggle
    d. Face shield
    e. Helmet (Cap)
    f. Foot wear (boots)
    g. Clothing/apron/overall.
    h. Disinfect equipment's and working area
9. If No Q7 why
    a. Lack of protective Equipment
    b. Negligence
    c. Not comfortable to use
    d. Lack of safety and health Education
    e. It decreases work performance
    f. not replace/new
    g. not size fit
    h. Lack of knowledge and practice
10. Do you eating well-cooked, Vitamin C content foods and safely handled meat
    A. Yes B. no
